# Subjective anxiety and depression in a sample of pregnant women with panic disorder

**DOI:** 10.1192/j.eurpsy.2025.2414

**Published:** 2025-08-26

**Authors:** I. Kamenova Ilieva, R. Vladimirova

**Affiliations:** 1 Psychiatry and Medical Psychology, Medical University Sofia, Sofia, Bulgaria

## Abstract

**Introduction:**

Panic disorder and depression during pregnancy are significant mental health concerns that can adversely affect both maternal and fetal well-being. Prompt recognition and management of these conditions through early screening are essential, not only for maternal health but also for the neuropsychological development of the child.

**Objectives:**

This study aims to assess anxiety and depression in pregnant women diagnosed with panic disorder, utilizing both objective and subjective measurement tools while integrating patient perspectives.

**Methods:**

The study included pregnant women with a confirmed diagnosis of panic disorder, evaluated in an outpatient setting (N=40). The participants were divided into three groups: (1) those with panic disorder (42.5%), (2) panic disorder with agoraphobia (20.0%), and (3) panic disorder with depression (37.5%). Objective measures such as the Hamilton Depression Rating Scale (HAM-D) and the Hamilton Anxiety Rating Scale (HAM-A) were used, along with self-reported scales, including the Hospital Anxiety and Depression Scale (HADS-A/HADS-D). Statistical methods, including descriptive analyses, the Student’s t-test, One-way ANOVA, and multiple linear regression, were employed. Ethical approval was obtained for the study.

**Results:**

Most participants were in their first pregnancy (77.5%) and had no prior psychiatric history (75%). Depression scores, measured by HADS-D (F(2, 37) = 6.05, p = .005, ω = .20) and HAM-D (F(2, 37) = 5.71, p = .007, ω = .19), were significantly higher in patients with depression compared to those with panic disorder, with or without agoraphobia. Higher gestational age was also associated with increased self-reported depression (p = .002, R² = .324, F(6) = 2.632, p = .034). Anxiety, as measured by the HADS-A scale, was significantly higher in the panic disorder group (F(2, 37) = 71.12, p < .01, ω = .78) than in the agoraphobia and depression groups. No significant differences were found between the groups on the HAM-A scale (p > .05).

**Conclusions:**

Proper identification of anxiety and depression during pregnancy is essential, as these conditions can negatively affect maternal functioning and quality of life. Moreover, they may hinder the mother’s ability to care for her infant postnatally. Utilizing both objective and subjective tools to assess anxiety and depression can improve diagnostic accuracy. Recognizing depression as a distinct domain in cases of anxiety disorder during pregnancy is also crucial for targeted intervention.

**Disclosure of Interest:**

I. Kamenova Ilieva Grant / Research support from: This study was supported by the National Scientific Program “Young Scientists and Postdocs,” funded by the Medical University of Sofia and the Ministry of Education and Science, Bulgaria., R. Vladimirova: None Declared

